# Efficacy of a single dose of milbemycin oxime/praziquantel combination tablets, Milpro^®^, against adult *Echinococcus multilocularis* in dogs and both adult and immature *E. multilocularis* in young cats

**DOI:** 10.1007/s00436-015-4855-7

**Published:** 2015-12-11

**Authors:** Dejan Cvejic, Claudia Schneider, Josephus Fourie, Christa de Vos, Stephane Bonneau, Natalia Bernachon, Klaus Hellmann

**Affiliations:** KLIFOVET AG, Geyerspergerstr. 27, 80689 Munich, Germany; Clinvet International Ltd, Bloemfontein, Republic of South Africa; Medical and R&D Department, Virbac SA, Carros Cedex, France

**Keywords:** *E. multilocularis*, *Echinococcus*, Dogs, Cats, Milbemycin, Praziquantel, Milpro®

## Abstract

Two single-site, laboratory, negatively controlled, masked, randomised dose confirmation studies were performed: one in dogs, the other in cats. After a period of acclimatisation, both the dogs and cats were orally infected with *Echinococcus multilocularis * protoscoleces. In the dog study, 10 dogs received a single dose of Milpro® tablets at a minimum dose of 0.5 mg/kg milbemycin oxime and 5 mg/kg praziquantel 18 days post-infection and 10 dogs received no treatment. In the cat study, 10 cats received a single dose of Milpro® tablets at a minimum dose of 2 mg/kg milbemycin oxime and 5 mg/kg praziquantel 7 days post-infection, 10 cats received a single dose of the treatment 18 days post-infection and 10 cats remained untreated. In both studies, intestinal worm counts were performed 23 days post-infection at necropsy. No worms were retrieved from any of the 30 treated animals. Nine of 10 control dogs had multiple worms (geometric mean 91, arithmetic mean 304) and all 10 control cats had multiple worms (geometric mean 216, arithmetic mean 481). The difference in worm counts between all three treated groups and their controls was highly significant (ANOVA *p* values of log transformed data <0.0001). Efficacy of 100 % was demonstrated for the elimination of adult *E. multilocularis* in dogs and cats as well as for elimination of immature *E. multilocularis* in cats as evidenced by the effectiveness of treatment 7 days post-infection. The treatments were well accepted and tolerated, and there were no adverse drug reactions observed.

## Introduction

*Echinococcus multilocularis* is one of the small cestodes of the *Echinococcus* genus in the family of *Taeniidae*, which has a widespread distribution in the northern hemisphere and whose known distribution is increasing in Europe and elsewhere (Eckert and Deplazes 1999; Romig et al*.*^1999^; Wahlstrom et al. 2011). The definitive hosts for this tapeworm are carnivores, generally canids (foxes, wolves, coyotes) and the principle wild definitive host in Europe is the Red Fox (Jenkins and Romig 2000), but felids can also act as definitive hosts (Deplazes et al. 1999, Jenkins and Romig 2000). Consequently both domestic dogs and cats can harbour patent infections (Dyachenko et al. 2008). There has been some debate about the role domestic cats may play in the epidemiology of the infection (Kapel et al*.*^2006^). *E. multilocularis* worms resulting from both, experimental and natural infections in cats can produce thick-shelled eggs, and therefore, cats should be considered as hosts and a source of potential infection (Thompson et al*.*^2003^). Due to the small size of the adult worms and their relatively small nutrient requirements, overt clinical signs associated with the parasite in the definitive host species are unusual, generally minor in nature (intestinal upsets) and are thought to occur only when the worm burdens are very high. In the definitive host, the developing and adult worms inhabit the small intestine and proglottids separate from the worm, pass through the bowel and are excreted in and around faecal matter. For completion of the lifecycle, mammalian intermediate hosts transmit the larval form of the parasite (i.e. metacestode) by ingesting embryonated eggs which develop into onchospheres that migrate through body tissues via blood vessels and then settle in organs. The metacestode then develops as a continuously growing tumour-like polycystic mass that is not clearly separated from the host tissues. Developed protoscoleces are found within these multiple cysts, which when ingested by the definitive host develop into adult worms. Many species, primarily small rodents, can fulfil this intermediate host role, e.g. mice, rats, voles, and shrews. Humans can be included in this list as a potential intermediate host, although the human being might be better described as a “dead-end or incidental host”, in that relatively few humans will be ultimately eaten by definitive hosts. However, the zoonotic risk posed by this species of worm is considerable, in that, ingestion of embryonated eggs passed by definitive hosts results in the development of alveolar echinococcosis in man (also known as alveolar hydatidosis), which is considered to be the most serious parasitic disease in humans in Europe (Romig 2009) with a prevalence of <1.4 per 100,000 population (Ammann et al*.*^2001^). In the absence of a successful treatment, which is difficult to achieve, the condition has a fatal outcome. Close social contact between humans and infected domestic dogs and cats is likely to be an increased risk factor for alveolar echinococcosis as demonstrated by Kreidl et al*.* (1998) who identified an associated increased risk with both cat ownership and hunting. Petavy et al*.* (2000) proposed that the prevalence of *E. multilocularis* in the domestic cat in an endemic area of alveolar echinococcosis is a risk factor for human beings, especially veterinarians. Consequently, despite not appearing to be overtly detrimental to the health of domestic dogs and cats, vigilant prevention of *E. multilocularis* infections in domestic dogs and cats in endemic areas together with surveillance, i.e. screening and monitoring the prevalence in certain wildlife species, is necessary in order to reduce the risks of human infection and further spread of the parasite. Additionally, dogs living in endemic areas of the *E. multilocularis*, having access to rodents, should be treated at 4-week intervals with an effective anthelmintic containing praziquantel or epsiprantel in order to prevent zoonosis (ESCCAP Guideline 01 Second Edition 2010).

Milpro® tablets are new formulations containing milbemycin oxime and praziquantel as active ingredients. Milbemycin oxime is a macrocyclic lactone. The efficacy of milbemycin oxime has long been established against intestinal nematodes, pulmonary and cardiac helminths including heartworm larvae and arachnids. It is ineffective as a treatment against cestodes.

Praziquantel, an acylated isoquinoline pyrazine compound, has been demonstrated to have cestocidal effects on both juvenile and adult worms for 40 years (Thomas and Andrews 1976; Thomas and Gonnert 1978). In cats and dogs, praziquantel at 5 mg/kg has proved effectiveness against both immature and adult *E. multilocularis* as well as many other species of cestodes (Thomas and Gonnert 1978). Praziquantel is effective at extremely low concentrations if it comes into contact with the parasite (Thomas and Andrews 1976). Praziquantel acts directly on contact with the parasites tegument and damages the structure (Markovki et al. 2006) as well as affects its carbohydrate metabolism and disrupts calcium homeostasis and membrane permeability to calcium ions in the parasite (Greenberg 2005) thereby resulting in tetanic contraction of the parasites’ musculature and subsequent tegumental disintegration.

The combination of milbemycin oxime with praziquantel does not reduce the efficacy of praziquantel against *E. multilocularis* (Jenkins and Romig 2003).

Although there are orally administered milbemycin oxime/praziquantel combination products with the same concentrations of actives approved for the veterinary market, there is the regulatory requirement to perform clinical efficacy studies. Because of the nature of *E. multilocularis* and the inherent zoonotic risk of working with this parasite, strictly controlled terminal laboratory studies using experimental infections have to be performed. For effective control of such a parasite it is essential to achieve 100 % efficacy.

### Materials and methods

Both studies were performed at the same laboratory in compliance with the International Cooperation on Harmonisation of Technical requirements for Registration of Veterinary Medicinal Products (VICH) guideline 9 for Good Clinical Practice (VICH 2001a). The studies followed also the applicable other VICH guidance documents: “Efficacy requirements for anthelmintics: Overall guidelines” (VICH 2000), “Effectiveness of anthelmintics: Specific recommendations for canine” (VICH 2001b) and “Effectiveness of anthelmintics: Specific recommendations for feline” (VICH 2001c). The protocol was subjected to review and satisfy both the sponsor’s and the laboratory’s animal welfare and ethical committees as well as satisfy the applicable national standard for “The care and use of animals for scientific purposes” (SABS 2008) and the local regulatory requirements.

Masking of personnel responsible for collecting assessment data to treatment allocation was enabled by the use of unmasked dispensers that played no other part in study procedures.

#### Animal selection

Both dogs and cats were vaccinated and de-wormed prior to pre-selection and the commencement of acclimatisation. The dogs underwent a clinical examination 4 days prior to infection on day 0, and the cats were clinically examined on day −14, 14 days prior to infection. These clinical examinations formed part of the selection process for continued participation in the study.

Dogs and cats were selected if:

- they had been acclimated to the study site for at least 7 days.
- they were clinically healthy as verified by a veterinarian during acclimatisation.
- they were older than 8 weeks of age.
- they were not pregnant.
- they had been de-wormed prior to acclimatisation, and they were free from any resident helminth infections as determined by gross and microscopic faecal examination prior to experimental infection.
- they were not fractious.

Although the animals were housed individually throughout the study, they were able to see and “interact” with conspecifics. Their faeces and pens/cages were observed throughout acclimatisation for evidence of helminth infection including a microscopic qualitative faecal flotation assessment during acclimatisation.

#### Animal welfare and safety assessments

Beside clinical examinations performed on dogs (day −19) and cats (days −25, −12 and −1), from the commencement of acclimatisation (day −25) until the end of the in-life phase of the study (day 5), general health observations (GHOs) were made daily for all animals. Multiple additional health observations were made on the days of infection and dosing. Animals were controlled for the occurrence of adverse events (AEs); an AE is defined as “any observation in animals that is unfavourable and unintended and that occurs after the use of a veterinary product or investigational veterinary product (Milpro®), whether or not considered to be product related” (VICH 2001a).

Macroscopic post-mortem examination of all animals was performed at the time of worm retrieval at necropsy.

#### Animal infection with protoscoleces

Food was withdrawn from both dogs and cats on the afternoon of the day prior to experimental infection. Approximately 1 h prior to experimental infection, the cats were provided with 10–15 g of soft food. The dogs and cats received their normal feed approximately 2 h after the oral administration of the protoscoleces.

The infective material for both studies was stored in an antibiotic saline solution and derived from European isolates less than 10 years old, which had been passaged through voles and/or gerbils. The percentage viability of the protoscoleces was evaluated microscopically, based on movement and flame cell activity. Based on the percentage viability, protoscoleces counts and the pre-defined dilution, the infective doses were prepared consisting of approximately 15,000 protoscoleces in 1.4 mL for each dog and approximately 20,000 protoscoleces in 3 mL for each cat. These infective doses were administered by syringing the solution into the back of the oral cavity. Immediately, after administration, the dogs and cats received a further 1.4 and 3 mL of saline, respectively, to ensure effective administration of the infective dose.

Animals were observed immediately after administration and at 1 h (± 10 min) post-infection to check that there was no regurgitation and/or vomiting of the infective doses.

The experimental infection was performed applying the method described by Jenkins and Romig (2000).

#### Animal randomisation to treatment groups

Twenty dogs and 30 cats were selected for further study participation on day −19. Eligible dogs and cats in excess of 20 and 30, respectively, were withdrawn from the study in a randomised fashion. Dogs were randomised to treatment groups on day −1 and cats on day −12. Animals were first separated by gender and then ranked within gender, from the highest to lowest body weight; ascending order of identification numbers was used for tie breaking. Animals were blocked in 10 replicates of two in dog, and 10 replicates of three in cat study so that there were two balanced treatment groups of 10 dogs and three balanced treatment groups of 10 cats.

#### Animal dosing

Dogs were dosed with tablets containing 2.5 mg milbemycin oxime and 25 mg praziquantel or 12.5 mg milbemycin oxime and 125 mg praziquantel and Milpro® film-coated tablets for small dogs and puppies and Milpro® film-coated tablets for dogs. Cats were dosed with Milpro® film-coated tablets for small cats and kittens (see Tables 1 and 2)

**Table 1:** Dosing table for dogs

Body weight (kg)	Number of small dog/puppy equivalent tablets (2.5mg milbemycin oxime/25mg praziquantel)	Number of dog equivalent tablets (12.5mg milbemycin oxime/125mg praziquantel)
0.5–1.0	1/2	–
>1.0–5.0	1	–
>5.0–10.0	2	–
>10.0–25.0	–	1
>25.0–50.0	–	2

**Table 2:** Dosing table for cats

Body weight (kg)	Number of Milpro® film-coated tablets for small cats and kittens (4mg milbemycin oxime/10 mg praziquantel)
0.5–1.0	1/2
>1.0–2.0	1

The doses administered were in accordance with the Summary of Product Characteristics (SPC) of the respective product using the body weights measured and recorded the day prior to dosing (day −1 for dogs and days −12 and −1 for cats). Tablets were administered at the back of the oral cavity over the base of the tongue and ensured that they were swallowed. Immediately, post-dosing the oral cavity was checked to see that the tablet(s) had been swallowed. Each animal was observed intermittently for the first hour post-dosing and then hourly (± 15 min) for up to 4 h.

#### Worm retrieval and counting

Food was removed from the pens/cages on the afternoon of day 4; on day 5, all animals were euthanized. After laparotomy, the small intestine was ligated proximal of the pylorus and distal of the ileocaecocolic junction. The small intestine was then removed in its entirety, incised longitudinally and placed in a warm water bath (approximately 37 ° C) for at least an hour. The intestine was then gently rinsed and rubbed between two fingers to dislodge worms that were still attached. Finally the mucosal lining was gently scraped using a microscope slide or suitable alternative to ensure that all worms were removed from the mucosal lining. The intestinal contents and all washings were passed through a sieve with aperture size of 75 μm. The residue collected in the sieve was gently back-washed into a container labelled with the animals’ identity, and the contents were preserved in 10 % formalin. After the samples had been collected a stereomicroscope was used for performing worm counts. The number of scoleces was considered to be the “worm count”.

Procedures for worm retrieval were performed on a treatment group basis, with thorough cleaning of facilities and equipment between groups in order to prevent the risk of “cross contamination”. The method applied was based on the one described by Jenkins and Romig (2000).

## Results

### Animal selection

Twenty-six dogs were pre-selected for the study and acclimatised to the study environment. Of the 26 dogs, 2 of them did not satisfy the inclusion criteria; 1 dog was listless and was excluded for health reasons, the other dog was found to be infected with *Trichuris vulpis* on day −20 when microscopic faecal examination was performed. Four dogs were withdrawn in a randomised fashion, leaving 20 dogs to be allocated to two treatment groups of 10 dogs. The ages of the selected dogs ranged from 2.8 months to 6.6 years; their body weights on day −1 ranged from 4 to 30 kg, and 14 of the dogs were females and six were males.

Forty cats were pre-selected for the study. At the commencement of acclimatisation on day −25, four cats were withdrawn for health reasons. On day −7, one cat showed diarrhoea; despite treatment, the cat died the following day (day −6). On day −2, one cat was not weight bearing on its left hind limb and was withdrawn from the study on day −1. During acclimatisation, no cat was identified having any helminth infections. Four cats were withdrawn on day −1 in a randomised fashion leaving 30 cats (21 males and 9 females) to be allocated to the three treatment groups of 10 cats. The ages of the selected cats ranged from 10 to 24 weeks. The body weights of the selected cats on day 6 ranged from 0.55 to 1.8 kg.

#### Animal welfare and safety assessments

No AE was recorded in the dog study. One cat was recorded to be thin on day −1. This cat was a young growing adolescent that had in fact gained 30 g body weight between day −12 and day −1. This cat had received Milpro® on day −11. There were no animal health problems in either study in the periods between experimental infection and dosing with Milpro®; therefore, no concomitant treatments were applied.

All cats enrolled received prophylactic medication for coccidiosis with Baycox® 5 % solution per os and Purbac® tablets per os. The dogs and cats not enrolled onto the study received appropriate veterinary care.

At post-mortem examination, no macroscopic abnormalities were evident in any of the 20 dogs and 30 cats, other than those strictly related to the parasitic infection.

#### Animal infection with protoscoleces

The experimental infection with *E. multilocularis* proved to be successful in both studies; all control animals barring one dog harboured multiple *E. multilocularis* worms on day 5. There were no reports of regurgitation/vomiting post-infection.

#### Animal randomisation to treatment groups

The randomisation programme resulted in well balanced treatment groups with respect to gender and body weight in both studies.

In the dog study, there were seven females and three males in each treatment group, and the arithmetic mean body weight for group 1 (untreated controls) was 11.05 kg and for group 2 was 12.24 kg. Despite the range of ages (2.8 months–6.6 years), the two treatment groups were well balanced with respect to age, with seven of the 10 Group 1 dogs being <5.3 months old and six of the Group 2 dogs being <5.3 months old.

In the cat study, there were seven males and three females in each of the three treatment groups, and the arithmetic mean body weights for each treatment group were 1.218 kg (median 1.195 kg) in the first group, 1.193 kg (median 1.175 kg) in the second and 1.232 kg (median 1.175 kg) in the third one.

#### Animal dosing

None of the 30 animals dosed (10 dogs and 20 cats) with Milpro® had any apparent reaction to the dosing procedure or the tablets themselves. The treatment was well accepted and tolerated; no tablets were rejected and dosing was complete and in accordance with dose rates indicated in the protocol and SPCs of the products.

#### Worm counts

No worms were found in any of the Milpro®-dosed animals. Multiple worms (exclusively *E. multilocularis*) were found in all of the control animals besides one dog (see Tables 3 and 4). The geometric mean of worm counts in control dogs was 91 (arithmetic mean was 304), and the geometric mean worm count in control cats was 216 (arithmetic mean was 481). The efficacy of the milbemycin oxime/praziquantel combinations proved to be 100 % against adult *E. multilocularis* infection in dogs and 100 % against adult and immature *E. multilocularis* infections in cats with the differences between all treated groups and their controls being highly significant at 0.0001 (ANOVA *p* value log transformed).

**Table 3:** *E. multilocularis* scoleces recovered from dogs

Parameter based on total number of protoscoleces	Group 1 (negative control) *n* = 10	Group 2 (adults) (milbemycin/praziquantel on study day 18) *n* = 10
Median	312.5	0
Minimum	0	0
Maximum	990	0
Geometric mean	91	0
Arithmetic mean	304	0
ANOVA test for superiority	*p* < 0.0001

**Table 4:** *E. multilocularis* scoleces recovered from cats

Parameter based on total number of protoscoleces	Group 1 (negative control) *n* = 10	Group 2 (adults) (dosed with Milpro® on Day −11) *n* = 10	Group 3 (juveniles) (Milpro® on Day 0) *n* = 10
Median	461.5	0	0
Minimum	5	0	0
Maximum	1470	0	0
Geometric Mean	216	0	0
Arithmetic Mean	481	0	0
ANOVA test for superiority	*p* < 0.0001 Group 1 compared to Group 2 or Group 3)		

## Discussion

Praziquantel either alone or in combination, in different formulations and when administered by a variety of routes, has been demonstrated to be highly effective in the treatment of a wide variety of cestode infections in dogs and cats (e.g. Thomas and Andrews 1976; Guralp et al*.*^1976^; Anderson et al*.*^1978^; Thomas and Gonnert 1978; Anderson et al*.*^1981^; Jenkins and Romig 2000; Jenkins and Romig 2003; Derbala et al*.*^2007^; Altreuther et al*.*^2009^; Papini et al*.*^2010^; Tuzer et al*.*^2010^; Barnett et al*.*^2013^, etc.). Despite its long-term use against *Dipylidium caninum* over a 40-year period, it does not appear that resistance has developed to the drug (Barnett et al*.*^2013^) with 100 % efficacy at 5 mg/kg, it is unlikely that there will be tapeworms surviving to pass on resistance. Vokral et al*.* (2012) have shown that the “rat tapeworm” (*Hymenolepis diminuta*), as a representative cestode, is not able to deactivate praziquantel and as such its biotransformation enzymes are not able to contribute to the development of resistance to praziquantel. As recently as 2015, the combination of milbemycin oxime and praziquantel has been reported to be the optimum choice for the treatment and prevention of helminth infections in kennel dogs under field conditions (Rinaldi et al*.*^2015^).

The studies reported here were designed to confirm the efficacy of a combination dose of 5 mg/kg praziquantel and 0.5 mg/kg milbemycin in dogs and 5 mg/kg praziquantel and 2 mg/kg milbemycin in cats against *E. multilocularis* infections. This necessitates experimental infections of the host. It has been reported that cats are not as suitable as experimental hosts for this parasite as dogs are (Anderson et al*.*^1981^) and that worm recovery in dogs was significantly greater than in cats (Thompson et al*.*^2003^). However, in the cat study reported here, all control animals had multiple worms, demonstrating reliable infection, and therefore, necessary to adequate *Echinococcus* control in cats living in endemic areas.

The *E. multilocularis* worm burden in subadult foxes is significantly higher than the one seen in adults (Hofer et al*.*^2000^). This suggests that previous exposure to the parasite may stimulate some immune response, effecting a degree of control. The existence of host humoral immune responses in helminths has been suggested by many studies such as those performed by Gallie and Sewell (1983) as well as by Hrckova et al. (2007)). Young, previously naive (with respect to *E. multilocularis* infection) animals were included in all treatment groups in both studies reported here. The genders, body weights and ages of the animals in each treatment group, in each of the studies, were reasonably balanced, enabling unbiased assessments of post-dosing worm burdens.

Neither of the two control groups was “dosed” with placebo. However, it is unlikely that the oral administration of a placebo tablet would have resulted in a “stress” reaction that would affect worm burden positively or negatively, thereby biasing results. The studies were essentially dose-confirmation studies of the Milpro® formulations, tested in a well-established artificial infection model; the authors, therefore, consider that there was no relevant bias introduced by not treating the control animals with placebo. All observations were done by fully masked personnel, in order not to introduce any further bias.

No adverse event occurred and no concomitant drugs prior to the act of euthanasia were used in the animals enrolled in the study. Drugs for the prophylactic treatment of coccidiosis were administered to all cats. The prophylactic use of a product to prevent coccidian-related disease is a standard treatment in young puppies in the research facility; these drugs are not considered to have any cestocidal effect and none of the cats that were experimentally infected had diarrhoea due to coccidiosis or any other cause which might have resulted in expulsion of worms. These facts together with the finding that all control cats had multiple worms after necropsy indicate that this concomitant treatment did not interfere with the validity of the study results.

The control of *Echinococcus* species of helminths is a particular challenge in part due to the zoonotic risks (referred to in the introduction, with respect to *E. multilocularis*) and the finding that they require higher doses of anthelmintics than some of the other species of cestodes commonly found in dogs and cats. Fortunately, praziquantel has a very wide therapeutic index at 5 mg/kg, which is the indicated dose and which is sufficient to achieve 100 % efficacy against *E. multilocularis* and *E. granulosus.* Efficacy of 100 % is not only desirable, especially with a parasite such as *E. multilocularis* but also necessary in order to avoid endemic infection and to reduce the risk of developing drug resistance. The studies reported here demonstrated 100 % efficacy of the praziquantel component of the combination product against adult *E. multilocularis* in dogs and cats.

No adverse event was reported in either study. Praziquantel at a minimum dose of 5 mg/kg was demonstrated to be safe as well as effective.

The prevalence of cestode infection is underestimated by gross and microscopic faecal examination (Adolph et al. 2011) and even though immunologically associated assays have been and are being developed (Deplazes et al. 1999) for the purpose of control, it is probably better to utilise a regular prophylactic anthelmintic programme, rather than rely on identification and diagnosis prior to dosing. Praziquantel, being component of the broad spectrum milbemycin/praziquantel combination used in these studies, serves this aim well, demonstrating 100 % efficacy against *E. multilocularis*, found in dogs and cats at the dose of 5 mg/kg.

## Conclusions

The experimental infection of both dogs and cats with *E. multilocularis* protoscoleces was successful.

A single oral dose of Milpro® film-coated tablets for dogs and Milpro® film-coated tablets for small dogs and puppies was demonstrated to be a safe and 100 % effective treatment against *E. multilocularis* in dogs when administered at a dose rate of 5 mg/kg indicated in the products’ SPCs.

A single oral dose of Milpro® film-coated tablets for small cats and kittens was demonstrated to be a safe and 100 % effective treatment against both immature (7 days post-infection) and adult *E. multilocularis* in young cats when administered at the recommended dose of 5 m/kg as indicated in the product’s current SPC for other tapeworm species.

The minimum dose of 5 mg/kg praziquantel used in these studies proved to be safe and 100 % effective against adult *E. multilocularis* in dogs and immature and adult *E. multilocularis* in cats.
